# Different frequencies to estimate bone mineral content from raw bioelectrical impedance data in adolescent soccer players: a critical analysis

**DOI:** 10.3389/fnut.2024.1524034

**Published:** 2025-01-22

**Authors:** Marcus Vinicius de Oliveira Cattem, Josely Correa Koury

**Affiliations:** Nutrition Institute, Rio de Janeiro State University, Rio de Janeiro, Brazil

**Keywords:** bioimpedance, bone mineral content, body composition, DXA, MF-BIA, phase angle

## Abstract

**Introduction:**

Skeletal muscle mass, body cell mass, total body water (TBW), and bone mineral mass (BMC) are components of fat-free mass (FFM), which conducts electrical energy due to its high water and electrolyte content. Multifrequency bioelectrical impedance analysis (MF-BIA) has been used to predict FFM, and studies have explored its application for quantifying BMC, a subset of FFM. However, the accuracy of the BMC predicted using MF-BIA depends on the methodological rigor of the frequency selection. This study examined the relationships between BMC and raw MF-BIA data at different frequencies.

**Methods:**

The MF-BIA (SECA 515®) device obtained raw bioelectrical data at 5, 50, and 500 kHz. BMC was quantified using dual-energy X-ray absorptiometry (DXA). Multiple linear regression models and bioelectrical impedance vector analysis (BIVA) were applied to evaluate whole-body and segmental BMC relationships.

**Results:**

Male adolescent soccer players (*n* = 149; 15.6 ± 0.6 years) participated in this study. Whole-body BMC (R^2^ = 0.522), and upper and lower limb BMC (R^2^ = 0.349) were best predicted at 5 kHz, while trunk BMC (R^2^ = 0.301) was best predicted at 50 kHz. BIVA revealed a leftward vector shift in participants with higher BMC quartiles. The calculated phase angle (PhA) was significantly higher in the highest BMC quartile for 5, 50, and 500 kHz in both upper and lower limbs (*p* < 0.05).

**Conclusion:**

These findings indicate that MF-BIA could be a supplementary tool for studying BMC in adolescent athletes. However, its utility is constrained by prediction and interpretation errors, emphasizing the importance of careful frequency selection.

## 1 Introduction

Bioelectrical impedance analysis (BIA) measures impedance, which is the opposition of frequency-dependent current flow. Fat-free mass (FFM) consists of lean soft mass (LSM) and bone mineral content (BMC). It is a compartment that contains water and electrolytes, primary conductors of electrical current in the body (1). Raw bioelectrical impedance data are resistance (R), related to fluids and ionic components; reactance (Xc), related to nonpolar components; and phase angle (PhA), related to cell functionality and integrity. These data are obtained from BIA devices, whether single- or multi-frequency (2–4) However, not all multifrequency devices provide R, Xc, and PhA values across different frequencies, making the critical use of frequency-dependent calculations difficult.

Predictive equations to estimate total body water (TBW), FFM, LSM, and fat mass (FM) have been validated with R and Xc as variables frequently obtained using single-frequency (SF-BIA, at 50 kHz) (5, 6). Multifrequency-BIA (MF-BIA) devices present frequencies ranging from 1 to 1,000 kHz. In frequencies below 50 kHz, the electrical current primarily passes through the extracellular water (ECW), which is adequate to estimate ECW. On the other hand, at frequencies above 50 kHz, the current can pass through the cell membrane, which is sufficient to estimate intracellular water (ICW) and TBW (7). Modern BIA devices estimate total BMC based on a constant proportion of minerals in FFM (8, 9) or calculate total BMC as the difference between predicted FFM and LSM. Unfortunately, many devices do not show the FFM and LSM predictive equations developed and used, making it difficult to validate them on other devices or populations (5, 10).

Adolescent soccer players seem to be a good model for studying bone health data because they are exposed to higher osteogenic stimuli (11–17), and soccer is the most popular sport among adolescents around the world. BMC may increase due to growth and physical training, with different degrees across body segments (18, 19). Athletes build bone mass in different body regions and at different rates depending on the type and intensity of their training (18, 20). BMC is positively correlated to muscle strength and may influence athletic performance and injury prevention (19). For this reason, periodic BMC assessment is advised, although it exposes the adolescents to radiation. Thus, it is important to explore complementary tools to study BMC.

Dual-energy X-ray absorptiometry (DXA) is a reference method for obtaining bone health data, such as BMC and bone mineral density (BMD). Despite its importance in sports, the use of this method involves high costs, trained professionals, and exposure to low-dose radiation (5, 21–23). Some authors have considered BIA as a technology capable of predicting BMC since the bone can conduct electrical current (24, 25). To our knowledge, there are four studies investigating BMC prediction using single frequency-BIA (23, 26–28), two using dual-frequency BIA (20 and 100 kHz) (9, 29), and five using MF-BIA (30–34), but their validity and reliability remain uncertain since the equations and frequencies used are not informed, suggesting that the frequencies may not be suitable for BMC prediction.

The present study aimed to investigate the relationships between BMC and raw MF-BIA data at different frequencies in male adolescent soccer players. To our knowledge, this is the first study to investigate whether different frequency values might be an additional source of bias in interpreting bone information using MF-BIA as a tool.

## 2 Materials and methods

### 2.1 Experimental design and participants

This was a cross-sectional study. The sample size was determined *a priori* using statistical software (G*Power, version 3.1.9.7) assuming: effect size = 0.3; *α* = 0.05; power (1 − *β* error probability) = 0.95. The estimated number of participants was 134 individuals. Male adolescent soccer players were recruited from first-division soccer clubs; they were training regularly and participating in competitions. Male adolescent soccer athletes were selected in order to avoid confounding variables due to differences in training sports modalities, such as mechanical loading patterns and sex. A total of 149 healthy male adolescent soccer players (aged 13 to 19.5 y), participated in the study. All participants were multiracial individuals and were considered biologically mature according to age at peak height velocity as described by Moore et al. (35).

Each participant underwent a single session of anthropometric measurements, MF-BIA, and DXA. In order to minimize potential confounding factors and ensure the reliability of the results, before the assessments, participants were advised to come in their training attire, remove all jewelry and metallic items, adhere to a fasting period of water and food for at least 4 h, and empty their bladder. Furthermore, they were instructed to refrain from engaging in physical exercise and consuming stimulant substances on the evaluation day. Participants’ compliance with the pre-study instructions was ensured through close supervision of the technical staff. The staff monitored and confirmed adherence to all preparatory guidelines. No athlete was excluded from the analysis, as all participants adhered to the study requirements.

Recruitment and data collection took place at the State University of Rio de Janeiro between September and November 2022, involving adolescent soccer athletes invited through contact with their technical staff.

All participants and their guardians received detailed explanations regarding the study’s procedures and protocols. Furthermore, they expressed their agreement by completing and signing an informed assent/consent form. The Ethics Committee of the Pedro Ernesto Hospital granted ethical approval for the study (CEP/HUPE 58284021.9.0000.5259), which was conducted by the Declaration of Helsinki.

### 2.2 Measurements

All measurements were performed between 9:00 AM and 1:00 PM. Participants were assessed by the same trained team, each researcher performing their respective functions.

#### 2.2.1 Anthropometric measurements

Body height (SECA-264® Hamburg, Germany) and weight (SECA-515® Hamburg, Germany) were obtained with an accuracy of ±50 g up to 100 kg for the scale and ± 2 mm for the stadiometer, according to Lohman’s recommendation.

#### 2.2.2 Multifrequency bioelectrical impedance analyses

A reliable hydration status assessment was obtained by instructing the volunteers not to exercise 24 h before the assessment, to go over an overnight fast, and to refrain from drinking 4 h before. BIA measurements were taken after a 5-min rest, with the participants in the supine position, in a thermo-neutral environment of 25°C.

Bioelectrical data (R and Xc) were obtained for whole-body and segments (upper limb, lower limb, and trunk) using octopolar MF-BIA (SECA-515® Hamburg, Germany) in 19 frequencies ranging from 1 to 1,000 kHz. Methodological details including hydration status were described by Cattem et al. (36). Segmental analysis was conducted on the right side of the body. PhA was calculated using the equation PhA = arc tan Xc/R x 180/*π* (37).

Frequencies below 50 kHz are deemed low and permit exploration of the extracellular environment, whereas those above 50 kHz are deemed high and can penetrate the intracellular environment. The frequencies (<50 kHz and > 51 kHz) were selected according to the following criteria: (1) more correlations among BMC, R, and Xc; (2) higher Pearson correlation coefficient values; and (3) the frequencies most cited in previous studies relating to BMC and raw BIA (Table 1).

**Table 1:** Multifrequency bioelectrical impedance device characteristics used in previous studies on bone mineral content.

References	MF-BIA device	Frequencies (kHz)
Dual frequency devices		
Lee et al. (8)	InBody 230	20 and 100
Liao et al. (27)	InBody 230	
Multifrequency devices		
Castro et al. (28)	InBody 720	5, 50 and 250
Fürstenberg and Davenport (29)	InBody 720	
Fürstenberg and Davenport (30)	InBody 720	
Patil et al. (31)	InBody 720	
Wang et al. (32)	InBody 770	

#### 2.2.3 Bioelectrical impedance vector analysis

The BIVA is based on the measurement of the raw bioelectrical data, which are R and Xc, normalized by the height (H) of the participants (R/H and Xc/H) (35–37). The bivariate 95th percentile of confidence limits (confidence ellipsis) of the experimental data is plotted in an R/H versus Xc/H graph (38–40). The correlation between R and Xc determines the ellipsoidal form of the bivariate probability distributions (38–41).

BIVA Software (42) was used to plot RXc graphs and to compare 95th percentile confidence ellipses according to quartiles of BMC for the whole-body, right upper limb, right lower limb, and trunk.

#### 2.2.4 Dual X-ray absorptiometry

BMC (g), FM (kg), and LSM (kg) were obtained for whole-body and segments (upper limb, lower limb, and trunk) using Lunar iDXA device (enCore 2008 version 12.20, GE Healthcare, WI, United States). BMC was used because bioelectrical data are related to quantities (mass) of mineral elements, which are electrical conductors. A skilled radiology technician performed the scan according to the manufacturer’s guidelines and international criteria (43). Participants underwent the scanning procedure supine, aligned with the central table’s longitudinal axis.

### 2.3 Statistical analysis

Data distribution was tested using the Kolmogorov–Smirnov test, and variables were represented with mean, standard deviation, and 95% confidence interval. Pearson correlations were conducted to examine the associations between bioelectrical data and BMC, considering the whole body and segments. Additionally, multiple linear regression analyses were performed using BMC as an outcome variable, with bioelectrical R and Xc data serving as independent variables.

For whole-body measurements, the models included R, Xc, age, and body mass, whereas for segmental measurements, only age was used as an independent variable. The objective of the multiple linear regression (MLR) was to assess the extent to which bioelectrical data (R and Xc, or R/H and Xc/H) predict variations in BMC, and to examine the strength, direction, and significance of the relationships.

The following MLR indexes were calculated: Beta coefficients (*β*), which represent the standardized effect of each independent variable; b coefficients, which indicate the change in the dependent variable per change unit in the independent variable; tolerance, which measures the independence of predictors; variance inflation factor (VIF), which assesses multicollinearity; multiple R, which indicates the overall fit of the model; adjusted R^2^, which accounts for the determination coefficient considering the number of predictors; and standard error of estimate (SEE), which estimates the model’s precision in predicting the dependent variables.

BIVA 95% confidence ellipses were drawn in R-Xc graphs in order to compare quartiles of BMC, using Mahalanobis’ distance (D), and to verify whether R, Xc and PhA could differentiate BMC in whole-body and segments.

PhA values were compared according to quartiles of BMC using one-way ANOVA and Bonferroni *post-hoc* test. STATISTICA 10 software (Stat Soft. Inc., Tulsa, OK, United States) was used for all analyses, and *p* < 0.05 was considered significant.

## 3 Results

The male adolescent soccer athletes were 15.6 ± 0.6 years (95%CI: 15.4–15.9), 67.6 ± 8.9 kg (95%CI: 66.1–69.0), 176 ± 7.3 cm (95%CI: 174.8–177.2), and training volume was 5.0 ± 1.8 h per week.

Body composition and bioelectrical data of participants are shown in Table 2. Pearson correlations (r) between bioelectrical data and BMC in the whole body, right upper limb, right lower limb, and trunk in the full range of frequencies from 1 to 1,000 kHz are presented in Table 3. R showed a consistent negative correlation with BMC and LSM across all frequencies. Considering Xc, 5 kHz was the low frequency with more associations. The high frequencies with more associations were 500, 750, and 1,000 kHz. And 500 kHz showed the highest r values among others.

**Table 2:** Body composition and raw bioelectrical data at 50 kHz for whole body and segments in male adolescent soccer athletes (*n* = 149).

	Whole body				Right upper limb				Right lower limb				Trunk			
	Mean	SD	95% CI		Mean	SD	95% CI		Mean	SD	95% CI		Mean	SD	95% CI	
DXA																
BMC (g)	3090.1	471.2	3013.9	3166.4	202.8	36.1	197.0	208.7	663.6	99.5	647.5	679.7	878.5	168.2	851.2	905.7
LSM (kg)	56.0	7.4	54.8	57.2	3.4	0.6	3.3	3.5	10.1	1.4	9.9	10.4	25.8	3.6	25.2	26.4
FM (kg)	8.8	2.3	8.4	9.2	0.5	0.1	0.5	0.5	1.8	0.5	1.7	1.9	3.3	1.2	3.1	3.5
BMC (%)	4.55	0.28	4.50	4.59	4.93	0.33	4.88	4.99	5.29	0.42	5.22	5.35	2.92	0.25	2.87	2.96
LM (%)	82.54	2.76	82.09	82.98	82.09	3.01	81.61	82.58	80.68	3.06	80.18	81.18	86.22	3.30	85.69	86.76
FM (%)	12.92	2.83	12.46	13.37	12.97	3.05	12.48	13.47	14.03	3.20	13.52	14.55	10.86	3.28	10.33	11.39
MF-BI_50kHz_																
R (Ω)	596.1	54.9	587.2	605.0	337.3	34.8	331.7	342.9	236.1	22.9	232.4	239.8	20.7	1.9	20.4	21.0
Xc (Ω)	65.3	7.0	64.1	66.4	35.3	3.7	34.7	35.9	29.2	4.0	28.5	29.8	3.2	0.4	3.2	3.3
PhA (°)	6.3	0.6	6.2	6.4	6.0	0.5	5.9	6.1	7.1	0.7	6.9	7.2	8.9	1.0	8.7	9.0
R/H (Ω/m)	339.6	37.9	333.5	345.8												
Xc/H (Ω/m)	37.2	4.4	36.5	37.9												

BI, bioelectrical impedance; BMC, bone mineral content; BMD, bone mineral density; CI, confidence interval; LSM, lean soft mass; H, height; PhA, phase angle; R, resistance; SD, standard deviation; Xc, reactance.

**Table 3:** Correlations among raw bioelectrical data at 1 to 1,000 kHz frequencies and bone mineral content in whole-body and segments in adolescent soccer players (*n* = 149).

Bioelectrical impedance frequency	Resistance (Ω)				Reactance (Ω)				Phase angle (°)			
(kHz)	Whole-body	Right upper limb	Right lower limb	Trunk	Whole-body	Right upper limb	Right lower limb	Trunk	Whole-body	Right upper limb	Right lower limb	Trunk
1	**−0.35**	**−0.43**	**−0.22**	**−0.24**	0.16	0.13	0.13	0.01	**0.39**	**0.39**	**0.30**	0.04
1.5	**−0.35**	**−0.43**	**−0.22**	**−0.24**	**0.19**	0.13	0.13	0.04	**0.44**	**0.44**	**0.31**	0.09
2	**−0.35**	**−0.43**	**−0.22**	**−0.24**	**0.21**	0.13	0.13	0.08	**0.46**	**0.45**	**0.31**	0.15
3	**−0.35**	**−0.44**	**−0.23**	**−0.25**	**0.23**	0.15	0.13	**0.16**	**0.47**	**0.47**	**0.31**	**0.27**
5	**−0.36**	**−0.47**	**−0.23**	**−0.26**	**0.24**	**0.16**	0.12	**0.29**	**0.48**	**0.48**	**0.30**	**0.45**
7.5	**−0.38**	**−0.45**	**−0.24**	**−0.27**	**0.23**	0.15	0.10	**0.34**	**0.48**	**0.48**	**0.29**	**0.53**
10	**−0.39**	**−0.46**	**−0.25**	**−0.30**	**0.20**	0.13	0.08	0.10	**0.46**	**0.47**	**0.26**	**0.23**
15	**−0.40**	**−0.47**	**−0.26**	**−0.31**	0.16	0.09	0.05	**0.29**	**0.45**	**0.47**	**0.25**	**0.57**
20	**−0.41**	**−0.48**	**−0.27**	**−0.33**	0.12	0.04	0.02	**0.26**	**0.44**	**0.46**	**0.23**	**0.55**
30	**−0.43**	**−0.49**	**−0.28**	**−0.35**	0.05	−0.04	−0.03	**0.22**	**0.41**	**0.43**	**0.19**	**0.54**
50	**−0.44**	**−0.50**	**−0.29**	**−0.38**	−0.04	−0.15	−0.09	**0.17**	**0.36**	**0.38**	0.14	**0.50**
75	**−0.45**	**−0.50**	**−0.30**	**−0.40**	−0.12	−0.24	−0.14	0.13	**0.31**	**0.32**	0.09	**0.44**
100	**−0.45**	**−0.50**	**−0.30**	**−0.40**	−0.17	−0.31	−0.17	0.11	**0.27**	**0.25**	0.06	**0.39**
150	**−0.45**	**−0.50**	**−0.29**	**−0.41**	−0.25	−0.39	−0.22	0.12	**0.19**	0.12	0.00	**0.31**
200	**−0.45**	**−0.50**	**−0.29**	**−0.41**	−0.30	−0.44	−0.26	0.10	0.11	−0.01	−0.05	**0.23**
300	**−0.45**	**−0.50**	**−0.29**	**−0.41**	−0.35	−0.49	−0.30	−0.05	−0.01	−0.21	−0.13	0.04
500	**−0.44**	**−0.49**	**−0.28**	**−0.41**	**−0.34**	**−0.50**	**−0.34**	**−0.25**	−0.12	**−0.36**	**−0.24**	**−0.18**
750	**−0.44**	**−0.49**	**−0.28**	**−0.42**	**−0.29**	**−0.49**	**−0.34**	**−0.30**	−0.14	**−0.39**	**−0.29**	**−0.18**
1,000	**−0.44**	**−0.49**	**−0.28**	**−0.44**	**−0.22**	**−0.48**	**−0.34**	**−0.32**	−0.11	**−0.38**	**−0.29**	**−0.17**

Values in bold presented significative correlations (*p <* 0.05).

Multiple linear regression analysis identifying the impact of bioelectrical data, R/H and Xc/H, age, and body mass on BMC in the whole body is shown in Table 4. In models considering R/H and Xc/H, the highest adjusted R^2^ was observed at 5 kHz, explaining 52.2% of the data variance. The inclusion of age increased the adjusted R^2^, with the best result at 5 kHz, accounting for 65.0% of the variance, compared to 63.5% at 50 kHz and 64.4% at 500 kHz. The inclusion of body mass increased the adjusted R^2^ to 86.5%, eliminating the bioelectrical variables in the model, and rendering the differences in frequencies excluded from the model.

**Table 4:** Multiple linear regression analysis models considering whole body bone mineral content (g) as dependent variable and raw bioelectrical data obtained at 5, 50, and 500 kHz frequencies.

Frequency	Models	β	b	*p*	Tolerance	VIF	Multiple R	Adjusted R^2^	SEE
Whole-body	*R/H and Xc/H*								
5 kHz	Intercept		5413.2	**0.001**			0.727	0.522	325.8
	R/H	−0.815	−9.2	**0.001**	0.794	1.260			
	Xc/H	0.406	68.4	**0.001**					
50 kHz	Intercept		5633.9	**0.001**			0.709	0.496	334.5
	R/H	0.863	−10.7	**0.001**	0.557	1.797			
	Xc/H	0.277	29.5	**0.001**					
500 kHz	Intercept		5904.9	**0.001**			0.681	0.456	347.4
	R/H	−0.689	−9.7	**0.001**	0.329	3.039			
	Xc/H	0.010	1.7	**0.001**					
Whole-body	*R/H, Xc/H and age*								
5 kHz	Intercept		3188.5	**0.001**			0.813	**0.653**	277.4
	Age	0.428	129.7	**0.001**	0.726	1.378			
	R/H	−0.609	−6.9	**0.001**	0.634	1.578			
	Xc/H	0.183	30.9	**0.004**	0.613	1.631			
50 kHz	Intercept		3171.7	**0.001**			0.810	0.649	278.7
	Age	0.449	136.0	**0.001**	0.762	1.312			
	R/H	−0.573	−7.1	**0.001**	0.426	2.346			
	Xc/H	0.053	5.6	**0.460**	0.471	2.125			
500 kHz	Intercept		3138.0	**0.001**			0.806	0.642	281.7
	Age	0.457	138.7	**0.001**	0.888	1.126			
	R/H	−0.565	−7.9	**0.001**	0.321	3.113			
	Xc/H	0.044	7.1	0.611	0.329	3.036			
Whole-body	*R/H, Xc/H, age, and body mass*								
5 kHz	Intercept		−1000.7	**0.007**			0.931	0.864	174.1
	Weight	0.839	44.2	**0.001**	0.294	3.405			
	Age	0.197	59.8	**0.001**	0.612	1.635			
	R/H	0.039	0.4	0.501	0.277	3.607			
	Xc/H	−0.010	−0.1	0.986	0.557	1.794			
50 kHz	Intercept		−953.0	**0.009**			0.931	0.864	173.7
	Weight	0.836	44.0	**0.001**	0.303	3.300			
	Age	0.188	57.1	**0.001**	0.613	1.632			
	R/H	0.006	0.1	0.920	0.255	3.928			
	Xc/H	0.038	4.1	0.388	0.470	2.126			
500 kHz	Intercept		−921.8	**0.010**			0.931	0.864	174.0
	Weight	0.931	43.8	**0.001**	0.316	3.168			
	Age	0.203	61.4	**0.001**	0.702	1.424			
	R/H	0.001	0.007	0.994	0.218	4.581			
	Xc/H	0.036	5.857	0.498	0.329	3.036			

H, height; R, resistance; SEE, standard error of estimate; VIF, variance inflation factor; Xc, reactance. Significative models were marked in bold.

Multiple linear regression analysis identifying the impact of bioelectrical data, R, and Xc, on BMC in segments is presented in Table 5. In models considering only R and Xc for the right upper limb, the highest adjusted R^2^ was observed at 5 kHz, explaining 34.9% of the data variance. The inclusion of age increased the adjusted R^2^, with the best result also at 5 kHz, accounting for 48.5% of the variance, compared to 46.8% at 50 kHz and 47.6% at 500 kHz. In models considering only R and Xc for the right lower limb, the highest adjusted R^2^ was found at 5 kHz, explaining 15.3% of the data variance. The inclusion of age increased the adjusted R^2^, with the best outcome at 500 kHz, accounting for 36.4% of the variance, followed by 5 kHz with 32.6% and 50 kHz with 32.6%. For models considering only R and Xc in the trunk, the highest adjusted R^2^ was observed at 50 kHz, explaining 30.1% of the data variance. When age was included, the best model was at 500 kHz, with an adjusted R^2^ of 55.9%, followed by 50 kHz at 54.5% and 5 kHz at 52.4%.

**Table 5:** Multiple linear regression analysis models considering limb bone mineral content (g) as the dependent variable and raw bioelectrical data obtained at 5, 50, and 500 kHz frequencies.

Frequency	*Adjustment variables*	β	b	*p*	Tolerance	VIF	Multiple R	Adjusted R^2^	SEE
Right Upper limb	*R and Xc*								
5 kHz	Intercept		331.9	**0.001**		1.250	0.598	0.349	29.2
	R	−0.643	−0.6	**0.001**	0.800				
	Xc	0.450	6.3	**0.001**					
50 kHz	Intercept		342.1	**0.001**		1.771	0.549	0.292	30.4
	R	−0.702	−0.7	**0.001**	0.565				
	Xc	0.313	3.0	**0.001**					
500 kHz	Intercept		312.0	**0.001**	0.163	6.117	0.503	0.248	31.3
	Xc	−0.503	−3.6	**0.001**					
Right Upper limb	*R, Xc, and age*								
5 kHz	Intercept		171.8	**0.001**			0.704	0.485	25.9
	Age	0.422	9.8	**0.001**	0.777	1.287			
	R	−0.486	−0.5	**0.001**	0.700	1.429			
	Xc	0.238	3.3	**0.002**	0.636	1.573			
50 kHz	Intercept		161.9	**0.001**		1.048	0.690	0.468	26.4
	Age	0.490	11.4	**0.001**	0.954				
	R	−0.391	−0.4	**0.001**					
500 kHz	Intercept		111.6	**0.001**		1.045	0.695	0.476	26.1
	Age	0.491	11.4	**0.001**	0.957				
	Xc	0.401	−2.9	**0.001**					
Right Lower limb	*R and Xc*								
5 kHz	Intercept		935.0	**0.001**		1.590	0.406	0.153	91.5
	R	−0.489	−1.8	**0.001**	0.629				
	Xc	0.418	15.3	**0.001**					
50 kHz	Intercept		964.0	**0.001**	1.000	1.000	0.293	0.079	95.5
	R	−0.293	−1.3	**0.001**					
500 kHz	Intercept		901.1	**0.001**	1.000	1.000	0.340	0.109	93.9
	Xc	−0.340	−16.2	**0.001**					
Right Lower limb	*R, Xc and age*								
5 kHz	Intercept		391.5	**0.001**		1.988	0.576	0.322	81.9
	Age	0.526	33.7	**0.001**	0.503				
	R	−0.248	−0.9	**0.001**					
50 kHz	Intercept		424.5	**0.001**	0.994	1.006	0.579	0.326	81.7
	Age	0.501	32.1	**0.001**					
	R	−0.255	−1.1	**0.001**					
500 kHz	Intercept		379.8	**0.001**	0.999	1.001	0.611	**0.364**	79.3
	Age	0.508	32.5	**0.001**					
	Xc	−0.321	−15.3	**0.001**					
Trunk	*R and Xc*								
5 kHz	Intercept		1268.7	**0.001**		1.182	0.492	0.231	147.5
	R	−0.435	−32.9	**0.001**	0.846				
	Xc	0.457	245.2	**0.001**					
50 kHz	Intercept		1355.4	**0.001**		1.313	0.558	**0.301**	140.6
	R	−0.609	−53.3	**0.001**	0.762				
	Xc	0.466	194.0	**0.001**					
500 kHz	Intercept		1554.7	**0.001**		1.014	0.455	0.196	150.8
	R	−0.382	−39.7	**0.001**	0.986				
	Xc	−0.206	−32.1	**0.001**					
Trunk	*R, Xc, and age*								
5 kHz	Intercept		265.8	0.085			0.731	0.524	116.0
	Age	0.613	66.4	**0.001**	0.778	1.286			
	R	−0.292	−22.1	**0.001**	0.799	1.252			
	Xc	0.143	76.7	**0.043**	0.658	1.519			
50 kHz	Intercept		351.1	**0.024**			0.745	0.545	113.5
	Age	0.562	60.9	**0.001**	0.770	1.299			
	R	−0.367	−32.1	**0.001**	0.644	1.553			
	Xc	0.179	74.6	0.013	0.606	1.650			
500 kHz	Intercept		298.7	0.042			0.754	0.559	111.7
	Age	0.616	66.7	0.001	0.953	1.049			
	R	−0.256	−26.6	0.001	0.945	1.058			
	Xc	−0.175	−27.3	0.002	0.983	1.017			

H, height; R, resistance; SEE, standard error of estimate; VIF, variance inflation factor; Xc, reactance. Significative models were marked in bold.

**Figure 1:**
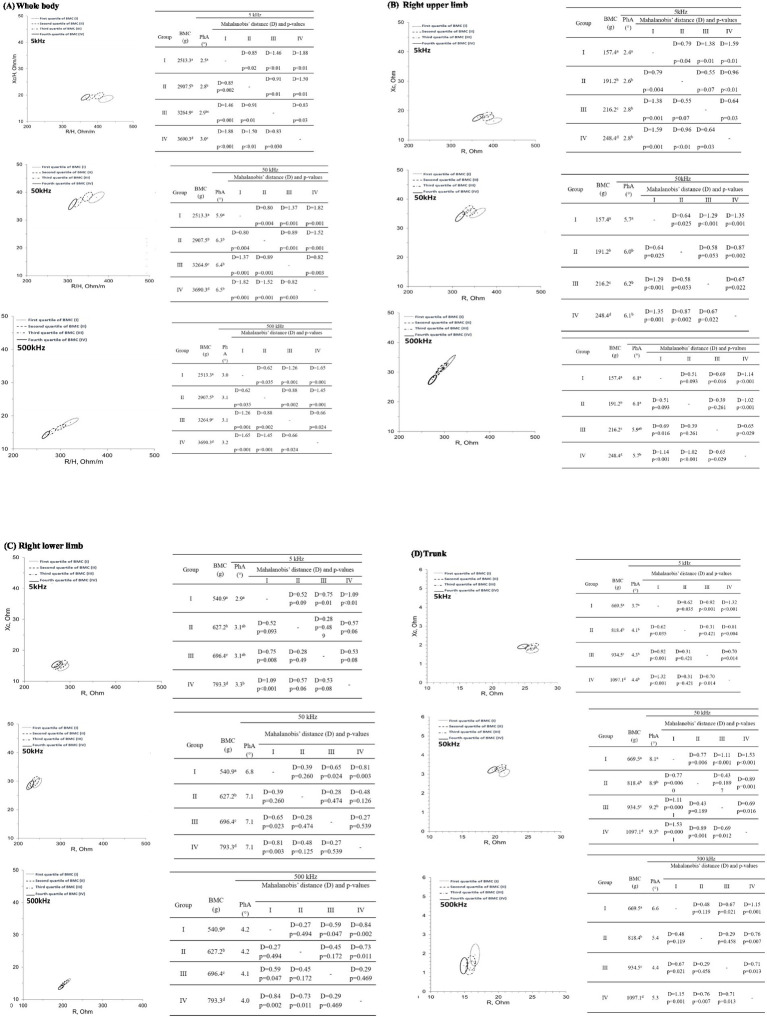
95% confidence ellipses considering **(A)** whole-body; **(B)** right upper limb bone; **(C)** right lower limb, and **(D)** trunk bone mineral content quartiles at 5, 50, and 500 kHz. BMC, bone mineral content; D, Mahalanobis’ distance; PhA, phase angle. Different letters in the same column indicate significant differences using one-way ANOVA and Bonferroni *post-hoc* test (*p* < 0.05).

BIVA confidence ellipses comparing quartiles of BMC are drawn for 5, 50, and 500 kHz, considering whole-body (Figure 1A), right upper limb (Figure 1B), right lower limb (Figure 1C), and trunk (Figure 1D). Additionally, the values of R/H and Xc/H for the whole body and R and Xc for the segments are provided according to the BMC quartiles. For all frequencies and segments (except trunk), the PhA value is higher in the fourth group (> BMC values) than in groups I, II, and III (*p* < 0.05).

## 4 Discussion

This is the first study that used an MF-BIA device to show that BMC values are related to raw bioelectrical data. The highest coefficient of determination of BMC for whole-body (52.2%), upper limb (34.9%), and lower limb (15.3%) was observed at 5 kHz. However, the coefficient of determination for the trunk was higher at 50 kHz (30.1%) than at 5 kHz (23.1%). This result may be related to the trunk’s body composition profile, which is approximately 50% of all LSM, whereas BMC is approximately 30% (8). This difference in proportion leads to the need for a higher frequency to conduct the electrical current.

The use of multiple frequencies allows for a more accurate assessment of the extracellular and intracellular environments, since low frequencies (<50 kHz) pass through the extracellular environment, and high frequencies (>50 kHz) penetrate the intracellular environment more effectively (1, 6, 7). Lower frequencies of electrical and dielectric properties of *ex vivo* distal femur and proximal tibia bones were investigated and pointed to mechanical properties and microdamage detection (24, 25). Overall impedance and resistivity seem to be better detectable at frequencies between 10 and 100 kHz, especially near the lower limit (44). The use of lower-intensity frequencies seems to be suitable for the assessment of BMC deposited in the bone matrix (24, 25, 44), however, ions in the LSM may be considered as confounding factors in *in vivo* measurements (8).

Some studies using BIA to investigate BMC prediction used only 50 kHz (23, 27, 28), 20 kHz, and 100 kHz (9, 29), or did not inform the specific frequencies used (30–34). The only study that developed an equation for BMC used the InBody720 analyzer as the reference method, thereby creating an indirect prediction, which accumulated bias (33). The present study demonstrated that the frequencies used are relevant for the BMC prediction and that lower frequencies are more representative of the BMC of the whole body and limbs when considering only bioelectrical data for prediction. Additionally, when age and body mass were included in the statistical model, they increased the coefficient of determination (R^2^) and removed raw bioelectrical data variables as predictors. Thus, using BIA to assess bone health without considering that the data are frequency-dependent could lead to additional errors in predicting or investigating bone characteristics using BIA.

In the present study, considering only models with bioelectrical data, 5 kHz frequency showed better results in explaining BMC and its classification into quartiles. At 5 kHz, vectors presented a better distinction of 95% confidence ellipses when comparing BMC quartiles, where the quartile with the highest BMC exhibited the highest PhA and the lowest R/H, and the quartile with the lowest BMC exhibited the opposite. These values can be explained by the higher amount of electrolytes in the extracellular environment, which increases electrical conductivity, conceptually opposite to resistance.

The limitations of the present study are related to the use of DXA because it does not provide information about bone quality or microarchitecture, which are critical determinants of bone strength. These parameters are influenced by body size and growth, potentially leading to underestimation or overestimation of BMC in adolescents with varying growth status. However, despite the limitations, DXA remains the preferred method for clinical measurements of bone density in children and adolescents because of its availability, reproducibility, speed, low exposure to ionizing radiation, and robust pediatric reference data.

Besides that, another limitation is that the participants were adolescent male soccer players, making it difficult to extrapolate our results to other populations. However, all participants were biologically mature according to peak height velocity, which reduced possible errors caused by groups with different maturity status.

The strength and most important finding of this study is that it was the first to consider body segment and show that BIA used to predict BMC must be taken with caution since frequency may influence the results. To our knowledge, no studies have a critical look at the use of BIA and its relation to bone variables.

Our results demonstrate a stronger association between the whole body, upper and lower limbs BMC, and bioelectrical impedance data at 50 kHz and 5 kHz, respectively. This reinforces the importance of selecting appropriate low frequencies when using BIA as a complementary tool for BMC studies. However, even with frequency optimization, our study highlights the limitations of BIA as an isolated tool for BMC prediction, further emphasizing the need for accurate methodologies in future research.

BIVA was able to identify the quartiles of BMC, proving its promising use in future studies in athletes from other sports modalities (high or low impact) since bone tissue is positively related to the impact. This approach could help establish MF-BIA as a complementary tool for monitoring bone health in diverse populations.

## 5 Conclusion

Our findings emphasize that the use of MF-BIA without clearly specifying the frequency could introduce significant bias in BMC estimation. Therefore, we discourage relying on BIA for obtaining BMC values in clinical or research settings, particularly when precise and reliable bone health assessments are required. These limitations highlight the importance of adopting rigorous methodologies and standardizing frequencies in future applications of BIA as a complementary tool in bone studies. Additionally, further research is essential to validate the reliability of BIVA as a complementary tool, particularly across diverse age groups.

## Data Availability

The data supporting this study´s findings are available on request from the corresponding author (JCK). The data are not publicly available due to their containing information that could compromise the privacy of research participants.
